# The Role of Epigenetics in the Pathogenesis and Potential Treatment of Obsessive-compulsive Disorder

**DOI:** 10.2174/011570159X347479241024120728

**Published:** 2025-02-12

**Authors:** Jacob Peedicayil

**Affiliations:** 1 Department of Pharmacology & Clinical Pharmacology, Christian Medical College, Vellore, India

**Keywords:** Epigenetics, obsessive-compulsive disorder, pathogenesis, treatment, quality of life, gene expression

## Abstract

Obsessive-compulsive disorder is a common neuropsychiatric disorder that markedly affects the quality of life of affected patients. There is increasing evidence that abnormal epigenetic mechanisms of gene expression are involved in the pathogenesis of this disorder. This article reviews the available data on epigenetic abnormalities found in patients with this disorder. The article also reviews the data on the use of epigenetic therapy in the treatment of obsessive-compulsive disorder, and epigenetic changes noted during psychotherapy of patients with this disorder. More detailed knowledge of the role of abnormal epigenetic mechanisms underlying obsessive-compulsive disorder could facilitate the development of new drugs for treating this disorder and the development of biomarkers for this disorder.

## 1 INTRODUCTION

Obsessive-compulsive disorder (OCD) is a neuropsychiatric disorder that is characterized by obsessions or compulsions, or both, which are distressing, time-consuming, or substantially impairing [1, 2]. Obsessions are recurrent thoughts and desires which are felt to be unwanted and that cause much discomfort. Compulsions are repetitive actions that the patient is compelled to do due to an obsession [2]. Obsessions and compulsions are time-consuming or cause marked difficulties in social, work, or other vital aspects of life [2]. The most common obsessions comprise a fear of contamination, fear of aggression or harm, fears about sex, fears about religion, and the need to make things “just correct” [2]. Over the past two decades, there has been a new field of research in biomedicine, including psychiatry, namely, epigenetics. Epigenetics, which means above or in addition to genetics, investigates changes in gene expression due to factors such as DNA methylation and histone modifications. There is accumulating evidence that epigenetics plays an important role in the pathogenesis of common mental disorders, including OCD.

To date, there have only been two review articles on the role of epigenetics in OCD [3, 4].

The present article updates the data given in those articles and discusses aspects of the role of epigenetics in the treatment of OCD not covered in them. It presents a narrative review of the role of epigenetics in the pathogenesis and potential treatment of OCD. The guidelines recommended by Murphy [5] for writing a review article were adhered to. The cited papers were obtained from PubMed and Google Scholar between January 1, 1988, and August 1, 2024. The search items employed were: DNA methylation, epigenetic or epigenetics, epigenetic therapy, epigenome-wide association study, histone, non-coding RNA, obsessive-compulsive disorder, pathogenesis, psychotherapy, and treatment. Articles reporting animal, human, cell line, and bioinformatics studies were included. Articles using languages other than English, and those reporting non-peer-reviewed data were excluded.

### 1.1 Epidemiology of Obsessive-compulsive Disorder

OCD is a common disorder and its lifetime prevalence is about 2% in the general population with similar rates across age groups and countries [6]. The average age of onset is 19.5 years with about half of those affected having the start of symptoms during childhood and adolescence [2]. OCD is more common in women than men in the general population with a gender ratio of 1.6:1 [7, 8]. There are some diseases related to and grouped with OCD. The related diseases include body dysmorphic disorder, hoarding disorder, trichotillomania (hair-pulling disorder), and excoriation disorder (skin-picking disorder) [1].

The clinical presentation of OCD is very heterogeneous. Unlike many other psychiatric disorders, where patients often present with similar symptoms, two patients with OCD only rarely share the same symptoms [6]. However, OCD symptoms do have commonalities connecting them to broader themes or symptom dimensions. There are four symptom dimensions with considerable support [6]: a) symmetry obsessions and repeating, ordering, and counting compulsions; b) disturbing thoughts, namely aggression, sexual, religious, somatic obsessions and checking compulsions; c) obsessions and compulsions related to cleanliness, contamination, and microbes; and d) hoarding obsessions and compulsions. Four novel dimensions of OCD are very common in daily clinical practice: a) transformation, namely, fears of turning into somebody or something else; b) body focus, that is, preoccupations with the body or somatic concerns; c) superstition, that is, magical thinking; and d)loss/separation, that is, fear of losing someone [6].

### 1.2 Biology of Obsessive-compulsive Disorder

As discussed above in Section 1.1, OCD is heterogenous in its clinical presentation. Not all patients respond to first-line treatments [9]. In this context, many neurocircuit models of OCD have been developed to enable a better understanding of the neural and cognitive processes implicated in the disease process. Such models employ developments in neuroscience and data from neuropsychological and neuroimaging studies to suggest connections between clinical profiles that mirror the symptoms and experiences of patients and malfunctions in specific neural pathways. It is possible that treatments for OCD can be made better if directed to malfunctioning of specific neurocircuits [9, 10].

For a long time, OCD has been thought to be a disorder of serotonin malfunction. The reason for this has been the fact that serotonin reuptake inhibitors such as selective serotonin reuptake inhibitors (SSRIs) and the tricyclic antidepressant clomipramine have established efficacy for the treatment of OCD [11]. Neuroimaging studies on the serotonin transporter and serotonin receptor density have given equivocal results, although they give some evidence of regional serotonin defects in the brain. These data are thought to be due to reduced synaptic serotonin in the cortico-striato-thalamo-cortical (CSTC) nerve tracts. Neuroimaging studies have also provided evidence of dopaminergic over-activity in the corpus striatum. There is also evidence of abnormalities in glutamatergic neurotransmission in the corpus striatum and GABAergic neurotransmission in the medial prefrontal cortex and the orbitofrontal cortex in OCD patients. The essence of OCD is thought to involve hyperactive CSTC neural pathways due to striatal dopaminergic and glutamatergic overactivity [11]. Vitamin D levels are low in patients with OCD, but it is at present not clear whether this is involved in the pathogenesis of OCD [12, 13].

Family and twin studies indicate that there is a genetic basis underlying OCD [14, 15]. OCD is a heritable disorder with contributions from common and rare variants including *de novo* mutations. Several candidate gene studies such as studies investigating genes associated with serotonergic, glutamatergic, and dopaminergic systems have not been successful [6, 7]. Genome-wide association studies (GWAS) on OCD are ongoing and have found many loci associated with the disorder, but the genetic basis of OCD is presently uncertain and more work needs to be done on this front [14]. Interestingly, whole genome association analysis of treatment response in patients with OCD suggests that genes underlying glutamatergic and serotonergic neurotransmission are associated with treatment response [16]. In addition to genetics, environmental stimuli contribute to the pathogenesis of OCD. Population-based [17], family-based [18], and twin-based [19] studies have shown that both genetic and environmental factors contribute to the pathogenesis of OCD. In a cohort of monozygotic (MZ) twins, some of whom were concordant for OCD and others discordant for OCD, it has been shown that stressful life events such as sexual abuse raise susceptibility to OCD [20, 21].

A subset of pediatric cases of OCD may be autoimmune or triggered by streptococcal infection. Such cases are referred to as pediatric autoimmune neuropsychiatric disorders associated with streptococcal infections (PANDAS) [22]. The development of PANDAS is thought to be sparked by streptococcal infection. Antibodies in response to group A β-hemolytic *Streptococcus* cross-react with self-autoantigens in the basal ganglia and cortical structures in the brain [22]. In this context, it is of interest that inflammatory changes have been noted in the brain in patients with OCD [23].

## 2 OUTLINE OF EPIGENETICS

Epigenetics, above or in addition to genetics, is presently a very active area of research in biomedicine. Epigenetics is often defined as the study of changes in gene expression not involving changes in DNA sequence. It refers to the regulation of gene expression by molecular mechanisms such as DNA methylation, DNA hydroxymethylation, histone modifications, and non-coding RNA (ncRNA)-mediated regulation of expression of genes. These molecular mechanisms interact with each other [24]. There is increasing evidence that epigenetic mechanisms of gene expression are dysregulated in diseases, especially in common, complex diseases [25]. Indeed, epigenetics has been called the epicenter of modern medicine [26] and has the potential to play a key role in preventing and treating disease [27].

## 3 THE ROLE OF EPIGENETICS IN OBSESSIVE-COMPULSIVE DISORDER

A lot of evidence suggests that epigenetic mechanisms of gene expression are involved in the pathogenesis of OCD: **1**. There is increasing evidence that dysregulated epigenetic mechanisms of gene expression take part in the pathogenesis of common mental disorders [28, 29]. **2**. The concordance rate of OCD among MZ twins falls short of 100%, suggesting the role of epigenetic mechanisms in OCD pathogenesis [30]. One study found that the concordance rate of OCD in MZ twins is only about 52% [31]. **3**. As mentioned above in Section 1.2, environmental factors play a role in the pathogenesis of OCD. Environmental factors can more easily act by modifying the epigenome rather than the genome [32]. Among environmental factors, non-shared environmental factors (environmental factors that make family members different from each other such as school friends and teachers) may be especially important in the pathogenesis of OCD [17, 19]. Some environmental factors may also be disorder-specific for OCD and related disorders [19]. **4**. There are sex differences among patients with OCD. For instance, there is evidence that male patients have an earlier age at the onset of symptoms and have a more protracted course of illness compared to female patients [33]. Male patients are also more likely to have a higher frequency of sexual and religious obsessions and greater social impairment [34]. Female patients more frequently have symptoms related to contamination and cleanliness and higher comorbidity with depression and suicidal risk [14]. These data could be explained by epigenetic abnormalities being involved in disease pathogenesis [35]. **5**. There are sex differences in the genetic architecture of patients with OCD. In this regard, Khramtsova *et al*. [36] characterized sex-specific genetic patterns across the genome in patients with OCD, using the biggest dataset of OCD patients and controls from the Psychiatric Genomics Consortium. The authors found two genes, GRID2 which encodes glutamate ionotropic receptor delta type subunit 2, and GPR135 which encodes G protein-coupled receptor 135, to be significantly associated with female patients with OCD but not male patients. The proteins encoded by these genes are both expressed in the brain. As for point number 4 given in this section above, these data could also be explained by epigenetic factors predisposing to OCD [35].

## 4 EVIDENCE FOR THE ROLE OF EPIGENETICS IN ANIMAL MODELS OF OBSESSIVE-COMPULSIVE DISORDER

Parvopassu and colleagues [37] conducted an interesting study that showed that epigenetic mechanisms of gene expression are involved in the development of OCD using the truncated dopamine transporter (DAT) rat model [37]. In this model the DAT gene has been knocked out, leading to increased dopamine in the synapse and dopaminergic overactivity. Truncated-DAT rats are an animal model for hyperactivity and compulsivity. The authors studied the developmental effects of the social environment on adolescent wild-type (WT) rats. At weaning (the time when the rats were started on solid foods), rats were divided into three groups: with another adolescent WT peer; with a WT adult rat; and with a truncated rat. The authors observed the home cage social behaviour of the pairs (that is, play, jump, victory, and bullying) during the whole of adolescence. When the rats became adults, the authors observed the same rats in plus maze, forced swim, and social preference tests for measuring levels of anxiety, depression, and quality of social interactions. It was found that in comparison to other groups, WT rats that had lived during adolescence with truncated DAT rats as companions showed more anxious, depressive, hyperactive, impulsive, and compulsive behaviour. The authors inferred from these data that a normal and healthy social environment is required for neurobehavioural maturation and that abnormal environmental interactions such as poor play and bullying may epigenetically affect the dopaminergic system causing possible signs of attention-deficit hyperactivity disorder and OCD.

Festucci and co-workers [38] used many protocols to study compulsive behaviour in rats. The authors put their focus on various epigenotypes related to the DAT. Compulsive behaviour was simulated by inflicting polydipsia with intermittent reward to the rats, causing them to drink large quantities of fluids. The authors used the Y-maze and marble-burying test to confirm the development of compulsive behaviour. There were differences between the different groups of rats according to epigenotype concerning compulsive behaviour. From their data, the authors conclude that the epigenetic context of the DAT gene affects the development of compulsive behaviour.

The endocannabinoid system (ECS) comprises endogenously formed molecules, the endocannabinoids, that bind to cannabinoid receptors [39]. The major endocannabinoids are signalling fats obtained from arachidonate and formed in cells all over the body, but they have been largely investigated in the nervous system [39]. The ECS is involved in the regulation of several key functions of the brain including emotional responses, control of posture and movement, learning and memory, inflammation, motivation to eat and sleep, and pain sensation [40]. The ECS has been implicated in the pathogenesis of many psychiatric disorders such as schizophrenia, bipolar disorder, and major depression [41].

The ECS may also be involved in the pathogenesis of OCD. In this regard, Bellia *et al*. [42] studied genes encoding enzymes involved in the biosynthesis of the endocannabinoids in blood samples of OCD patients and brain tissues of DAT-heterozygous rats showing compulsive behaviours. The authors found decreased levels of diacylglycerol lipase-α (DAG-α) and N-acyl phosphatide ethylamine-phospholipase D hydrolase (NAPE-PLD) in the amygdala complex and prefrontal cortex of DAT-heterozygous rats demonstrating compulsive behaviours. Additionally, mRNA levels of the genes encoding cannabinoid receptors types 1 and 2 were reduced in the amygdala and blood of OCD patients. It was also found that there was increased DNA methylation of the promoter of the gene encoding NAPE-PLD.

## 5 LABORATORY EVIDENCE FOR THE ROLE OF EPIGENETICS IN PATIENTS WITH OBSESSIVE-COMPULSIVE DISORDER

There have been several anecdotal and case reports of vitamin B_12_ (cyanocobalamin) deficiency and an excess of homocysteine in patients with OCD [43-46]. Balandeh *et al*. [47] recently conducted a well-designed systematic review and meta-analysis of serum vitamins and homocysteine levels in OCD patients. From their work, it was learnt that low levels of serum vitamin B_12_ and high levels of serum homocysteine significantly correlate with the symptoms of OCD in affected patients. These data are of interest regarding the role of epigenetics in OCD because vitamin B_12_ is required for the conversion of homocysteine to methionine. Methionine is converted to S-adenosylmethionine, the most important donor of a methyl group in most biochemical reactions in the body, including DNA methylation [48, 49]. Hence, a deficiency of vitamin B_12_ leads to lower levels of DNA methylation and higher levels of homocysteine [50].

Several studies have investigated epigenetic anomalies underlying OCD and to date, most have focused on changes in DNA methylation (Table **1**) [51-63]. These studies have found changes in the levels of DNA methylation in genes encoding the OXTR [53, 56], SERT [54], and brain-derived neurotrophic factor (BDNF) [55, 59]. Among these studies, the findings of Guo and colleagues [62] are interesting because these authors found differentially methylated positions (DMPs) and regions (DMRs) in OCD patients and comparison to control subjects. The genes in which the DMPs and DMRs are located include genes involved in immune system development, cerebellar cortex development, and regulation of nervous system development. As mentioned above in Section 1.2, inflammatory changes have been noted in the brain in patients with OCD [64].

However, the results of epigenetic studies are at present inconclusive since the levels of DNA methylation vary between studies. There are also inconsistencies among the findings. For instance, Grünblatt *et al.* [54] found raised DNA methylation levels in the gene encoding SERT in early-onset OCD patients in comparison to controls, whereas there was reduced DNA methylation in adult OCD patients in comparison to controls. Additionally, these authors found unchanged peripheral SERT gene expression levels despite higher DNA methylation levels in this gene. Moreover, two studies [55, 59] found DNA hypomethylation of the BDNF gene. This epigenetic change is known to typically cause an increase in gene expression, which would lead to higher peripheral levels of BDNF. However, a meta-analysis of BDNF levels in OCD patients found reduced peripheral levels of BDNF [64]. Other epigenetic abnormalities such as those involving histone modifications and ncRNAs have also been studied but again at present, the data is unclear partly because the studies are limited in number (Table **2**) [65-68].

## 6 THE ROLE OF EPIGENETICS IN THE TREATMENT OF OBSESSIVE-COMPULSIVE DISORDER

Epigenetic therapy, the use of epigenetic drugs to correct epigenetic abnormalities underlying disease, is a new development in pharmacology [69]. Dar *et al.* [70] conducted a comparative study of augmentation of SSRI therapy with the antipsychotics aripiprazole and olanzapine, and the epigenetic drug L-methyl folate, a methyl donor, that can methylate DNA. The study consisted of an open-label phase, 12 weeks long to determine treatment resistance to SSRIs. This was followed by another 6-week, open-label phase for non- or partial responders during the first phase. 115 patients entered the first phase. 60 patients entered the second phase where they received in addition to SSRIs, an antipsychotic or L-methyl folate. It was found that the patients who received antipsychotics demonstrated a significant improvement but there was no change in the patients who received L-methyl folate. A possible explanation for the lack of effect of L-methylfolate in the OCD patients in this study is that there is already DNA hypermethylation in several genes in OCD patients (Table **1**). N-acetylcysteine (NAC) is an antioxidant and also has epigenetic effects including upregulating sirtuins (class III histone deacetylases) [71]. NAC has been tried in the treatment of OCD. Two randomized double-blind controlled studies found NAC to be effective in the treatment of OCD [72, 73] whereas another did not [74]. Recently, the World Federation of Societies of Biological Psychiatry and the Canadian Network for Mood and Anxiety Disorders Task Force “weakly recommended” NAC at a dose of 2 to 3 grams per day for adjunctive use in the treatment of OCD [75].

There is increasing evidence that epigenetic mechanisms of gene expression play a part in psychotherapy [48, 76]. This endorses Nobel laureate Eric Kandel [77] who, as early as 1998, suggested that psychotherapy helps patients by altering gene expression in the brain, thereby causing changes in synaptic function and new structural brain changes. Several studies have investigated the potential role of changes in epigenetic mechanisms during psychotherapy of patients with OCD (Table **3**) [78-82]. These studies used cognitive behaviour therapy (CBT) and investigated epigenetic changes in blood samples from study patients. The studies so far have focused on DNA methylation and have noted DNA methylation changes in genes including those encoding monoamine oxidase-A (MAO-A), SERT, and OXTR (Table **3**). The study of Campos Martin *et al*. [82] is of interest since these authors conducted a robust two-step epigenome-wide association study (EWAS) in 185 OCD patients and 199 controls. This study enabled the detection of 305 differentially methylated CpG sites. The authors then authenticated these data by determining the best group of CpGs that could properly classify the study subjects into their respective groups. This led to the identification of 12 CpGs that overlapped with the 305 CpGs found in the EWAS. These 12 CpGs are close to or within genes linked with abnormal dopaminergic neurotransmission. However, the association of these 12 sites with responses to CBT only reached a trend level. We are at present in the early stages of research on the role played by epigenetics in the psychotherapy of OCD patients. In this regard, as noted by Syed and Zannas [83] more work needs to be done on the relationship between psychotherapy and changes in epigenetic mechanisms of gene expression before these data can be translated into patient care. For instance, the issue of whether peripheral tissues such as blood can be used as proxies for brain tissues has to be sorted out [83].

## CONCLUSION

The available data suggests that abnormal epigenetic mechanisms of gene expression contribute to OCD pathogenesis. However, more work needs to be done to understand what epigenetic changes are involved and in which genes epigenetic changes occur. It appears that there are changes in epigenetic mechanisms of gene expression during psychotherapy in patients with OCD. A better knowledge of the role of abnormal epigenetic mechanisms in OCD could facilitate the development of epigenetic drugs for the treatment of OCD and the development of biomarkers to diagnose OCD and assess the response of patients with OCD to treatment of this disorder.

## Figures and Tables

**Table 1:** Epigenetic (DNA methylation) changes found in patients with obsessive-compulsive disorder.

**Gene(s)**	**Sample Source**	**Finding**	**References**
ESR1, GABA-B1, MOG	Blood	DNA methylation associates with OCD in female adolescents	[51]
Several genes	Blood	DNA methylation associated with OCD	[52]
OXTR	Blood	Increased DNA methylation correlates with severity of OCD	[53]
SERT	Saliva	Increased DNA methylation in OCD patients	[54]
BDNF	PBMC	Hypomethylation of BDNF gene	[55]
OXTR	Leukocytes	DNA methylation negatively correlates with OCD	[56]
Several genes	Saliva	Different DNA methylation patterns	[57]
Several genes	Brain	DNA methylation associates with OCD	[58]
BDNF	Saliva	Hypomethylation of BDNF gene	[59]
EWAS	Blood	DNA methylation in several genes associates with OCD	[60]
OXTR	Blood, saliva	Interplay between microbiota and epigenetics of OXTR	[61]
Many genes	Peripheral blood cells	Many genes had DMPs and DMRs compared to controls	[62]
FKBP5	Leukocytes	Sex-dependent association with OCD	[63]

**Abbreviations**: BDNF = Brain-derived neurotrophic factor; DMP = Differentially methylated position; DMR = Differentially methylated region; ER1 = Estrogen receptor1; EWAS = Epigenome-wide association study; FKBP5 = FK506-Binding Protein 5; MAO-A = Monoamine oxidase- A; MOG = Myelin oligodendrocyte glycoprotein; OCD = Obsessive-compulsive disorder; OXTR = Oxytocin receptor; PBMC = Peripheral blood mononuclear cells; SERT = Serotonin transporter.

**Table 2:** Epigenetic (histone and non-coding RNA) changes found in patients with obsessive-compulsive disorder.

**Sample Source**	**Finding**	**References**
Blood	Association between HDAC2, HDAC3, HDAC4, and HDAC10 and OCD	[65]
Blood, Lymphoblast-Derived Cell lines	*De novo* mutations are enriched in genes regulating chromatin modification	[66]
Blood	Association of Anril, a lncRNA, with OCD	[67]
Bioinformatics database	Association of Hsa-miR-3121 and has-miR-495-3P with OCD	[68]

**Abbreviations**: HDAC = Histone deacetylase; lncRNA = Long noncoding RNA; miR = microRNA; OCD = obsessive-compulsive disorder.

**Table 3:** Epigenetic findings during psychotherapy in patients with OCD.

**Type of Psychotherapy**	**Epigenetic Change**	**References**
CBT	Increase in MOA-A gene DNA methylation	[78]
CBT	DNA methylation changes in *PIWILI*, *MIR886*	[79]
CBT	Low DNA methylation of SERT gene predicts poor response to CBT	[80]
CBT	Increased DNA methylation of OXTR gene predicts poor response to CBT	[81]
CBT	DNA methylation changes in genes underlying dopaminergic function	[82]

**Abbreviations**: CBT = Cognitive-based therapy; MOA = Monoamine oxidase; OXTR = Oxytocin receptor; SERT = Serotonin transporter.

## LIST OF ABBREVIATIONS

BDNF: Brain-derived Neurotrophic Factor
CBT: Cognitive Behaviour Therapy
CSTC: Cortico-striato-thalamo-cortical
DAT: Dopamine Transporter
DMPs: Differentially Methylated Positions
ECS: Endocannabinoid System
GWAS: Genome-wide Association Studies
MZ: Monozygotic
OCD: Obsessive-compulsive Disorder
SSRIs: Selective Serotonin Reuptake Inhibitors
WT: Wild-type
